# Improving the swelling capacity of granular cold-water rice starch by ultrasound-assisted alcoholic-alkaline treatment

**DOI:** 10.1016/j.ultsonch.2023.106506

**Published:** 2023-06-26

**Authors:** Kannika Kunyanee, Kanyarak Phadtaisong, Jutarat Na Chiangmai, Natch Parittapongsachai, Tai Van Ngo, Naphatrapi Luangsakul, Sirada Sungsinchai

**Affiliations:** School of Food Industry, King Mongkut’s Institute of Technology Ladkrabang, Bangkok 10520, Thailand

**Keywords:** Granular cold-water swelling starch, Rice starch, Ultrasound assisted, Physical properties

## Abstract

The aim of this study was to determine the ability to improve the capacity of cold swelling and cold-water solubility of rice starch by ultrasonic-assisted alcohol-alkaline and alcohol-alkaline methods. To achieve this, ultrasound powers (U) were varied (30%, 70%, 100%) under the granular cold-water swelling starch (GCWSS) preparation (GCWSS + 30 %U, GCWSS + 70 %U, and GCWSS + 100 %U). The effects of these methods on morphological, pasting properties, amylose content, ratio of 1047/1022 spectra by FTIR, turbidity, freeze–thaw stability, and gel texture were also studied and compared. The results showed that the surface of GCWSS granules presented a honeycomb especially GCWSS + U treatments exhibited more porous on the surface of starch granules. The cold swelling power and solubility of GCWSS + U samples were increased which confirmed by reducing ratio of ordered structure to amorphous structure of starch, and turbidity was also decreased. Moreover, pasting temperature, breakdown, final viscosity, and setback decreased while peak viscosity increased as measured using a Rapid Visco Analyzer. The freeze–thaw stability of GCWSS + U was more resistant to syneresis than GCWSS under repeated freeze–thaw cycles. The reduction of gel hardness and springiness was observed using Texture Analyzer. These changes were enhanced with increasing ultrasound powers. Thus, the results indicate that the different ultrasound-assisted alcohol-alkaline treatments for preparing GCWSS show an effective use in the preparation of GCWSS with improved cold-water swelling and reduced retrogradation of rice starch.

## Highlights

• Granular cold-water swelling starch (GCWSS) with ultrasound treatment decreased the amylose content of rice starch and changed the pasting properties.
• GCWSS with aid of ultrasound could quickly swell into cold water.
• Ultrasound treatment generated porous on the surface of the GCWSS granules and enhanced water absorption increased.
• Ultrasound treatment decreased turbidity and retrogradation of GCWSS starch.

## Graphical abstract

**Figure:**
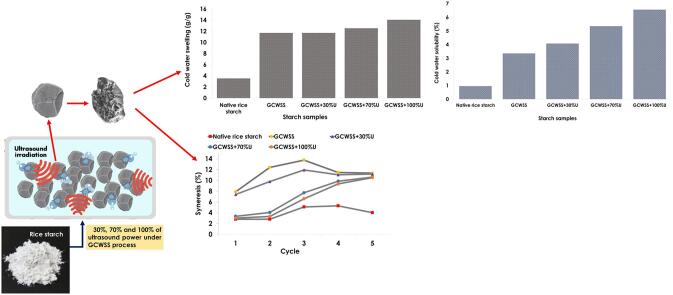

## 1 Introduction

Starch is one of the natural polymer compounds found abundantly in the world, which is also widely used in the food industry due to possessing many distinct functional properties [1], [2]. Starch contains two main components, amylose and amylopectin, which give a semi-crystalline structure and make up the insolubility of starch granules at room temperature [3]. Therefore, natural starch requires energy to gelatinize and dissolve in water. Generally, the heating process at 60–70 °C increases the movement of starch molecules and causes swelling of the granules, later the disruption of intermolecular interaction liberates the hydroxyl group and leads to an increase in binding to the water molecule by hydrogen bonding [4], [5]. Thus, due to this requirement, the application of the native form was limited, especially for the production of instant foods and also foods containing heat-sensitive materials as bioactive compounds [6]. As a result, in recent years there has been a lot of interest in changing the properties and structures of native starches into a soluble and swell form at room temperature, as known as granular-cold-water-swelling starch, that improve utilization of these starches in food sector [1], [7], [8].

Granular-cold-water-swelling starch (GCWSS) has a highly amorphous structure which may potentially increase the hydroxyl groups' accessibility to water. Therefore, this starch can rapidly and largely dissolve in cold because of its high-water absorption and swelling without heating assistance [9]. GCWSS is used as a thickening ingredient in puddings, sauces, salad dressings, and instant foods such as dry mixes, dry soups, and infant food water. Additionally, it is utilized in non-thermal food processing to create microwaveable foods, contain bioactive ingredients, use heat-sensitive colorants, and are cold desserts [10]. The GCWSS starches are produced using a variety of techniques, including heating starches in aqueous alcohol, high temperature, and pressure conditions, and alcoholic-alkaline treatment [7], [10], [11], [12]. Physical modification, either alone or in combination with chemical reactions, has been extensively studied and applied to change the structure of starch molecules and increase the functional properties of the native starches. However, consumers nowadays are more considering about the safety of the product. Since GCWSS manufacturing involves physical modulation, it can be utilized without restriction in food production as an ingredient, particularly in the creation of products with clean and green labels [10]. A physical procedure called ultrasonication uses ultrasound waves at frequencies above the range of human hearing. The process of ultrasonication, which is also known as “green modification” alters the structure and characteristics of starch in a starch–water mixture by locally producing significant shear forces, high temperatures, and free radicals [13], [14]. In recent years, the process of ultrasound-assisted alcoholic-alkaline treatment for granular cold water swelling maize starches was performed and optimized [15], [16]. These studies have shown that ultrasound assisted treatment can not only alter some of the functional properties of maize starches but also improve the solubility until approximately 75% [16]. Recent report of Majzoobi and Farahnaky [10] showed that corn starch was mainly researched by scientists. While it is still limited in the number of research to understand the mechanism effect of sonication process on GCWSS in different starch sources. Furthermore, rice starch also showed wide range of application as well as potential to produce cold water swelling starch as shown in the research of Butt, Ali [17]. Therefore, this study aimed to comprehensive investigate the effect of the aid of ultrasound waves on the production of GCWS under alcoholic-alkaline treatment, especially on rice starch, which has not been studied yet. It is possible that rice starch’s structural changes brought on by the ultrasonic-assisted alcoholic-alkaline treatment could help to increase its solubility in cold water. The findings of this study will be helpful for further optimizing and upscale production in the food industry with one of abundant source as rice starch.

## 2 Materials and methods

### 2.1 Materials

Commercial rice starch was purchased from Thai Flour Industry Co., Ltd. (Nakhon Pathom, Thailand). Protein, fat, and amylose content of native rice starch was 1.31%, 0.01%, and 19.14%, respectively. Ethanol (99% purity), hydrochloric acid (HCl), and sodium hydroxide (NaOH) were purchased from RCI Lapscan, Ireland.

### 2.2 Sample preparation

Granular-cold water swelling rice starches were prepared by the method of Zhu, Liu [16] with slight modifications. 100 g of rice starch (dry weight basis) was suspended in 40% w/w ethanol solution (800 mL) at ambient temperatures with continuous mixing using a magnetic stirrer (Remi Motors, India) at 450 rpm for 10 min. Sodium hydroxide solution (3 M, 200 g) was followed to add slowly (approximately 4 mL/min) to the prepared suspension with gentle stirring. Then, the mixture suspensions were placed in an ultrasound bath for 30 min (WUC-D10H, Wisd, Daihan Scientific, Korea) at the operation with 30% (GCWSS + 30 %U), 70% (GCWSS + 70 %U), and 100% (GCWSS + 100 %U) of ultrasonic power (665 W, 60 kHz). The temperature during ultrasound treatment was controlled at 25℃. This suspension was centrifuged at 5000 rpm for 10 min. The liquid phase was decanted and resuspended with 500 mL of 40 %w/w ethanol solution. Then, the pellet was neutralized with HCl (3 M in ethanol). The neutralized starch was washed with 300 mL of 60% and 99.9% ethanol solution, respectively. This suspension solution was centrifuged at 5000 rpm for 10 min. The obtained starch was dried in a hot air oven at 50℃ to a moisture content of 11%. The dried starch was milled and screened pass through a sieve 100 mesh size, placed into a zip-lock bag and stored at room temperature until analysis. Rice starch was treated with alcoholic-alkaline treatment without an ultrasound process and estimated in the same way. This sample was labeled “GCWSS”.

### 2.3 Scanning electron microscopy (SEM)

The starch sample was sprayed on an aluminum stub with double-adhesive tape. The sample stage was sputter-coated with gold. After that, the sample was observed using FEI Quanta scanning electron microscope (250-FEG, USA) under a vacuum at the accelerating voltage of 20 kV.

### 2.4 Apparent amylose content

The amylose content of rice starch and GCWSS samples was determined by an iodine colorimetric method described by Fang, Huang [18]. Briefly, 5 mL solution of urea dimethyl sulfoxide (UDMSO) was used to dissolve 10 mg of starch (exactly 0.1 mg) in the tube. The dissolving process involved an hour incubation period at 95 °C with intermittent vortexing. The starch-UDMSO solution was diluted to a level of 50 mL with water after being treated with a 1 mL aliquot of iodine solution (0.2% I_2_ and 2% KI, w/v). The mixture was quickly mixed and left in the dark for 20 min. The absorbance at 620 nm was recorded to calculate the apparent amylose concentration. By using a standard curve made using potato amylose (Sigma-Aldrich), the recorded data were converted to percent of amylose.

### 2.5 Cold-water absorption and cold-water solubility

The cold-water absorption and cold-water solubility of GCWS rice starch were measured according to the method of Hedayati, Shahidi [19] with slight modifications. 0.1 g of starch samples were weighed and added with 10 mL of distilled water. The mixture was instantly stirred using a voltage mixer for 30 sec. The suspension was then continuously mixed at an ambient temperature for 30 min, then centrifuged at 4500 rpm for 20 min. The supernatants were poured into a pre-weigh aluminum can and dried in a hot air oven at 105℃ to a constant weight. The cold-water absorption and cold-water solubility were calculated by the following equation:

Cold-waterabsorption(g/g)=(residueweight/weightofstarch)×100.

Cold-watersolubility(%)=(driedsupernatantweight/weightofstarch)×100.

### 2.6 Fourier transform infrared spectroscopy (FTIR)

Fourier transform infrared spectroscopy (FTIR) (Bruker, Germany) was used to measure the starch samples' FTIR pattern. OPUS 8.0 software was used to baseline correct and normalize the spectra, which were collected in the 4000–400 cm^−1^ range. They computed the intensity ratio (R value) at 1047 and 1022 cm^−1^ (1047/1022, R), which as known as ratio of ordered structure starch to amorphous structure starch [18].

### 2.7 Pasting properties

The pasting properties of starches were measured using Rapid Visco Analyzer (RVA) (model 4, Newport Scientific, Australia) following the method of Approved Method 61–02 (AACC, 2000). 3 g of Rice starch was weighed and added 25 g of distilled water into an RVA canister. The suspension was heated to 50℃ with stirred at 160 rpm for 1 min, heated to 95℃ at the rate of 5℃/min for 3.42 min, held at 95℃ for 2.3 min, cooled to 50 °C, and finally held at 50℃ for 3 min. The pasting profiles were recorded including pasting temperature (PT), pasting viscosity (PV), breakdown (BD), and final viscosity (FV).

### 2.8 Turbidity measurement

The analysis of turbidity of starches was a modified version of Ye, Luo [20]. A 1% aqueous suspension of rice starch was heated at 95℃ in a water bath for 1 h with continuous mixing and cooled rapidly to 30℃. The turbidity was measured by determining the absorbance at 640 nm (A640) using a UV–visible spectrophotometer (UV-1800, Shimadzu, Japan). Distilled water was used as a blank, the A640 value of which was subtracted from the A640 value for the solution containing starch.

### 2.9 Freeze-thaw stability measurement

The method of freeze–thaw stability was determined according to the method described by Hedayati, Shahidi [21] with slight modification. The starch sample was added into a 50 mL centrifugal tube with distilled water at the ratio of 1:9. The tube was stored at −20℃ for 24 h followed by thawing at 30℃ for 1 h in a water bath. After that, the tube was centrifuged at 4500 rpm for 10 min. The supernatant was removed and weighed. This sample was repeated up to five cycles. The percentage of water syneresis was calculated as the equation:

Water syneresis (%) = (weight of the water separated/the initial gel weight) × 100.

### 2.10 Gel texture analysis

After pasting analysis, the starch paste in a container was sealed with paraffin film and kept for 24 h at 4℃. The gel was then analyzed texture profile using Texture Analyzer (TA-XT2i) following the method of Wu, Han [22]. The textural properties were determined 24 h after the gel preparation. The textural properties were analyzed including gel hardness and springiness, which recorded directly by the texture profile curve on analysis system. The operation was controlled with a deformation level of 25% and crosshead speed was 50 mm/min and fitted with a cylindrical probe (35 mm diameter). The hardness is related to the strength of gel structure under compression and is the peak force during the first compression cycle. Springiness is related to the height that the food recovers during the time that elapses during the end of first bite and the start of the second bite [23].

### 2.11 Statistical analysis

All the samples were carried out in at least three replicates and the data water analyzed using SPSS for window version 21 (SPSS Institute Inc., Chicago, IL, USA). Analysis of variance (ANOVA) was performed, and Duncan’s multiple range test was used for comparison of means at a significance level of 0.05.

## 3 Results and discussion

### 3.1 Morphological properties

**Fig. 1:**
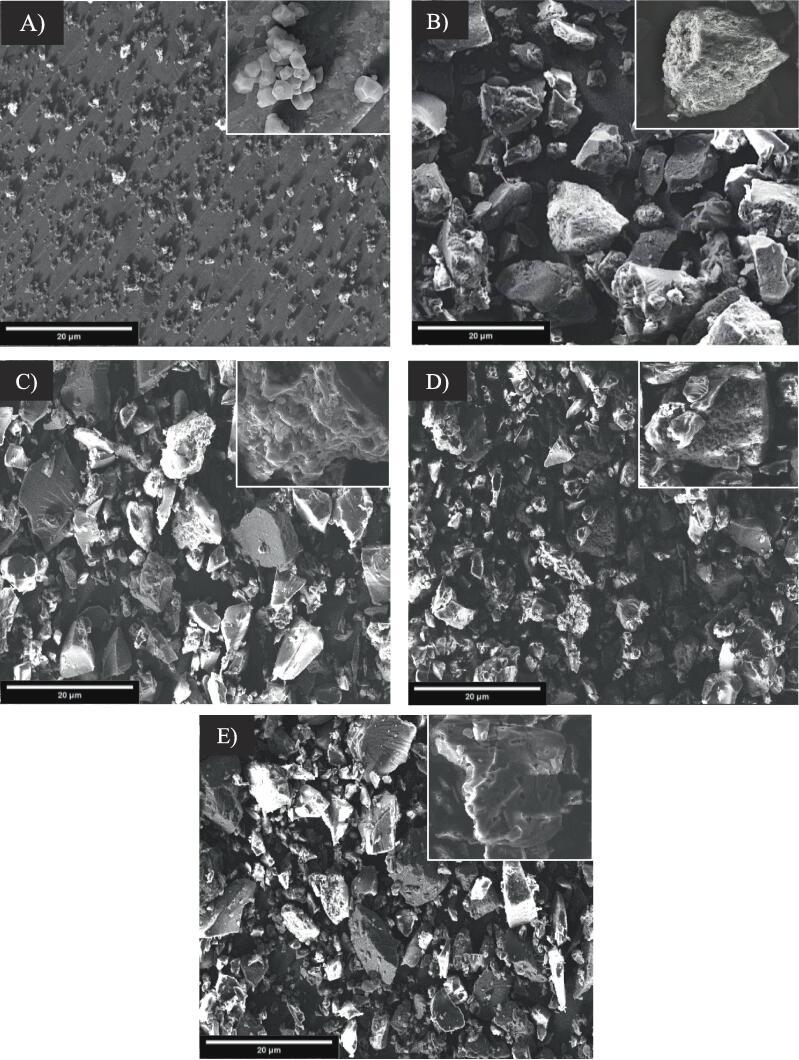
Scanning electron micrographs of the native rice starch (A), GCWSS (B), GCWSS + 30 %U (C), GCWSS + 70 %U (D), and GCWSS + 100 %U (E).

In the process of changing rice starch swelling and solubility characteristics by alcoholic-alkaline treatment with/without aid of ultrasonic waves, the appearance structure of starch granules could change. The scanning electron micrographs (SEM), one of the structure analysis methods, shows the surface difference between the native rice starch and GCWSS granules under different treatment (Fig. 1). SEM micrographs showed that the native rice starch granules displayed mainly polyhedral shape and smooth surface without cracks (Fig. 1A). Meanwhile, an indented and glommed appearance of the granules was found in the GCWSS rice starch. Additionally, GCWSS sample also showed the granules swollen and larger in size after alcoholic-alkaline treatment. This result due to the function of the alkali induces the electrostatic repulsion between negatively charged dissociated hydroxyl group of starch molecules and consequently swelling and deformation of granules [11], [24]. However, the combination of alcoholic-alkaline treatment with ultrasound wave (GCWSS + U samples) led to a significant change in the structure of rice starch, which is presented in Fig. 1C-E. The increase in ultrasound power treated GCWSS exhibited increased number of individual particles with a deformed shape and the surface of the granules become rougher with some fissures. Especially, GCWSS + 100 %U sample can be seen honeycomb with appearance several pits on some granules. The cavitation impact and mechanical effect of the ultrasonic treatment mainly caused a change on the surface of the GCWSS rice starch. Similarly the result by the finding of Bai, Hebraud [25] reported that potato starch surface displayed pitting, shallow fissures on potato starch granules after being treated with a higher power of ultrasound treatment.

### 3.2 Amylose content

The amylose content of native rice starch and GCWSS samples are shown in Table 1. All GCWSS rice samples had amylose content between 21.99 and 26.64%, which was higher than the native rice starch (19.14% amylose content). The increased amylose content of GCWSS might be due to the degradation of amylopectin chains to form short chains amylose chains during modification using alcoholic-alkaline treatment [26]. Kaur, Fazilah [27] reported a similar result for sago starch, which has a higher amylose content after alcoholic-alkaline treatment. Additionally, amylose content was decreased by ultrasound treatment with a higher power level, decreasing from 26.64% to 21.99% which was lower than the GCWSS treatment. This demonstrated that the starch granules might have disrupted and destroyed inter- and intra-starch molecules by ultrasound treatment, resulting in the leaching of amylose out of the granules and producing the reduction of amylose content [10], [28]. This result was similar with that of Abedi, Pourmohammadi [29] found that tapioca starch ultrasound-assisted gelatinization enhanced the reduction of amylose content.

**Table 1:** Amylose content, cold water absorption (g/g) and solubility (%) of native, GCWSS and GCWSS treated with ultrasound treatment.

**Starch sample**	**Amylose content** **(%d.b.)**	**Cold water swelling**	**Cold water solubility**
		**(g/g)**	**(%)**
Native rice starch	19.14 ± 0.93^e^	3.54 ± 0.05^c^	0.96 ± 0.54^d^
GCWSS	26.64 ± 0.18^a^	11.71 ± 0.96^b^	3.36 ± 1.09^c^
GCWSS + 30 %U	25.42 ± 0.37^b^	11.72 ± 0.88^b^	4.08 ± 0.98^bc^
GCWSS + 70 %U	24.23 ± 0.23^c^	12.54 ± 1.24^ab^	5.35 ± 0.25^ab^
GCWSS + 100 %U	21.99 ± 0.57^d^	14.07 ± 0.04^a^	6.56 ± 0.29^a^

Different letters in the same column indicate significant difference from each other *(p* ≤ 0.05).

### 3.3 Cold water swelling and cold water solubility

Water swelling and water solubility are indicators of the interactions between the starch molecule chains in the crystalline and amorphous regions with water molecules. Cold-water swelling and cold-water solubility of the native rice starch and GCWSS samples are also shown in Table 1. Upon alcoholic-alkaline treatment, GCWSS sample had significantly different higher water swelling (3.3–3.9 times) and water solubility (3.5–6.8 times) than the native rice starch (*p* ≤ 0.05) at a room temperature. This result could be due to the function of the alkaline producing starch molecules negatively charged, which led the starch granular swell by the forces of electrostatic repulsion mechanism. As swelling develops, double helical regions become single helical regions, and the crystalline structure is disrupted, resulting in amylose molecules being leached out of starch molecules; thus, the solubility value of GCWSS increased. Meanwhile, when comparing GWCSS samples, samples with ultrasonic treatment showed remarkably higher swelling and water solubility at room temperature than untreated samples. Specifically, the water swelling of the GWCSS + U samples was higher about 0.09–20.15% compared with the GWCSS sample when the ultrasonic amplitude power increased from 30% to 100%. Besides that, the cold-water solubility capacity also enhanced significantly by 21.4–95.2% when applying ultrasound waves combined with alcoholic-alkaline treatment. This result may be due to the influence of granular surface change as previously analyzed results by SEM. The mechanical jet impact of ultrasonic waves that create cavitation collapse of bubbles on the surface leading to cracks and fissures surface that increase the permeability of water into the starch granules [15]. The increased water penetrability is more pronounced due to starch granules containing pores characteristic resulting in the starch granules being easily swollen [14]. Since the ultrasound wave disrupts the surface of the granules, it might be exposed to a critical step in producing a higher percentage of short-chain starch molecules and facilitating it more able to absorb and disperse water, increasing water absorption [29], [30]. Furthermore, as the ultrasound wave disrupts the crystalline structure which contribute to amylose and/or amylopectin short chains are easy to leach out of the granule; thus, the water solubility also greatly increased. This result corresponds to the finding of Zhu, Liu [16].

### 3.4 Fourier transform infrared spectroscopy (FTIR)

**Fig. 2:**
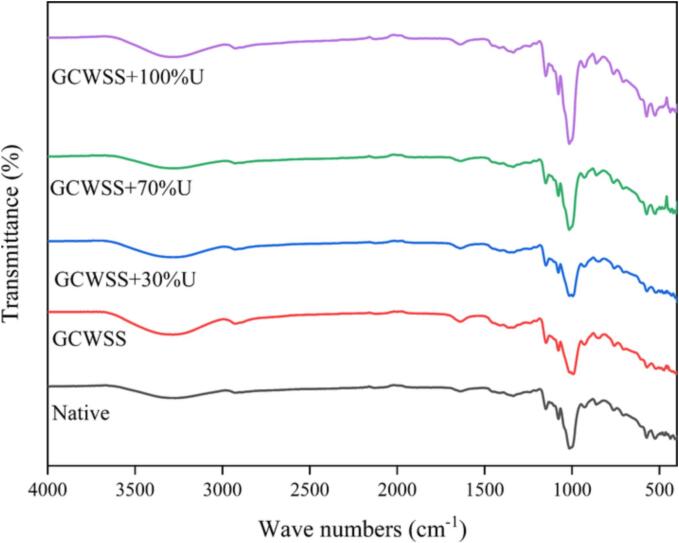
FTIR spectrum of the native, GCWSS and GCWSS + U samples.

Fig. 2 illustrates the FTIR spectra of the native, GCWSS, and GCWSS + U samples. The positions of the characteristic absorption peaks remain largely unchanged following both alcoholic-alkaline treatment and ultrasonic-assisted alcoholic-alkaline treatment. This lack of change suggests that these treatments, whether applied individually or in combination, do not lead to the formation of any new compounds. The ratio of the height of absorbance bands at 1047 cm^−1^ (C = C stretching) and 1022 cm^−1^ (C = O stretching), known as the infrared index R1047/1022 (R value), serves as an indicator of the amount of ordered starch compared to amorphous starch [17]. When starch is subjected to the ethanol-alkali process and further treated with ultrasound, the R value decreases. This reduction in the R value is dependent on the intensity of sonication. The R values for native starch, GCWSS, and GCWSS + U are measured to be 0.7219 ± 0.0111, 0.6984 ± 0.0035, and ranging from 0.6789 ± 0.0007 to 0.6934 ± 0.0081, respectively (Table 2). Higher ultrasound power corresponds to lower R values, indicating a decrease in the presence of short-range ordered structure within the starch samples in GCWSS + U. Upon modification, a shift in the spectrum around the 980–1020 cm^−1^ peak was observed, particularly in GCWSS samples treated with ultrasound power ranging from 70% to 100%. This shift can be attributed to the disruption of intra-molecular hydrogen bonds in rice starch caused by ultrasound, leading to changes in spectral patterns and increased starch solubility [7]. Our findings suggest that both single and dual treatments result in a reduction in the compactness of starch granules, thereby increasing the ability of water to penetrate the starch matrix [18]. This corroborates the previous observation of increased cold-water absorption and swelling.

**Table 2:** Intensity ratio (R value) at 1047 and 1022 cm^−1^ of native, GCWSS and GCWSS treated with ultrasound treatment by FTIR.

**Starch sample**	**R value**
Native rice starch	0.7219 ± 0.0111^a^
GCWSS	0.6980 ± 0.0035^b^
GCWSS + 30 %U	0.6934 ± 0.0081^b^
GCWSS + 70 %U	0.6894 ± 0.0013^bc^
GCWSS + 100 %U	0.6789 ± 0.0007^c^

Different letters in the same column indicate significant difference from each other *(p* ≤ 0.05).

### 3.5 Pasting properties

The pasting properties of native rice starch and GCWSS rice starch samples are summarized in Table 3. The pasting profiles are related to viscosity, stability, and retrogradation of starch pastes [31]. The native rice starch, GCWSS and GCWSS + U samples showed significantly different (*p* ≤ 0.05) in pasting temperature, peak viscosity, breakdown, and final viscosity. GCWSS sample showed a significantly lower pasting temperature, pasting viscosity, breakdown, and final viscosity, while exhibited a higher setback value as compared to the native rice starch. These results could be due to the destruction of crystalline structure during alcoholic-alkaline treatment, resulting in decreased gelatinization temperature and pasting viscosity of starch paste [32]. Additionally, a higher of setback value of GCWSS might be related with their amylose content increased which reflects a rapid aggregation of amylose chain during cooling cycle [33]. GCWSS + U samples presented decreased the pasting temperature from 71.65 to 63.70 °C. This result could be due to starch granules had damaged by the ultrasound treatment enhancing its viscosity at gelatinization temperatures below [15]. GCWSS + U with increasing ultrasound power samples showed a higher peak viscosity than GCWSS sample. This is positively related to the increase in swelling capacity of GCWSS + U samples (as presented in Table1) as an effect of ultrasound waves. It could be possibly indicated that while the alcoholic-alkaline treatment modified the crystalline structure of starch granular, the addition of higher ultrasound power could cause more degradation of starch molecules and/or attributed to weakening of the linkages between amylose and amylopectin led to a greater water absorption contributed to starch swollen, resulting in a higher viscosity of GCWSS + U [34]. Breakdown value represents the tendency of starch pastes during gelatinization process, as known as starch paste stability [35]. The decrease in breakdown value of GCWSS + U samples indicates that these samples have less paste stability and more resistant to shear thinning during heating [35]. Although, ultrasound treatment could produce damaged starch granules. However, ultrasound waves also assisted to produce the higher percent of short-chain starch facilitating for retrogradation process to form into perfect crystallite and more organized configuration of double helices [30]. Setback value is referred to a short-term retrogradation of amylose during cooling process. The final viscosity and setback values of GCWSS + U samples were significantly lower than GCWSS. Additionally, a higher ultrasound power treated GCWSS sample exhibited a significantly decrease in setback value, these results indicated that ultrasound treatment could destroy the amorphous structure of starch molecules during treatment, and it could contribute more damage of amylose chains, which might affect the rearrangement of amylose in cooling process [36]. This suggested that the improvement solubility and swelling capacity of rice starch by alcoholic-alkaline plus ultrasound treatment can reduce the retrogradation and the formation of gels.

**Table 3:** Pasting properties of the native, GCWSS and GCWSS treated with ultrasound treatment.

**Sample**	**Pasting temperature** **(°C)**	**Pasting profiles (Rapid Visco Unit, RVU)**			
		**Peak viscosity**	**Breakdown**	**Final viscosity**	**Setback**
Native rice starch	89.60 ± 0.27^a^	218.78 ± 0.15^a^	31.26 ± 0.06^a^	223.65 ± 0.55^a^	40.98 ± 0.37^e^
GCWSS	71.65 ± 0.28^b^	76.42 ± 0.11^e^	28.41 ± 0.11^b^	130.30 ± 0.18^e^	68.80 ± 0.92^a^
GCWSS + 30 %U	68.55 ± 0.06^c^	88.91 ± 0.04^d^	23.71 ± 0.04^c^	168.76 ± 0.36^b^	61.34 ± 0.44^b^
GCWSS + 70 %U	67.10 ± 0.02^d^	100.39 ± 0.09^c^	23.21 ± 0.02^d^	155.52 ± 0.24^c^	57.06 ± 0.42^c^
GCWSS + 100 %U	63.70 ± 0.19^e^	104.33 ± 0.07^b^	19.52 ± 0.08^e^	153.60 ± 0.59^d^	52.12 ± 0.87^d^

Different letters in the same column indicate significant differences from each other *(p* ≤ 0.05).

### 3.6 Turbidity measurement

Turbidity measurement is another approach used to evaluate starch retrogradation [37], which a lower transmittance value indicates a higher turbidity of starch paste [38]. Turbidity of the native rice starch, GCWSS and GCWSS + U samples were reported in term of the percentage of transmittance (% Transmittance) as shown in Table 4. The transmittance values were in the range of 33.53% – 40.23%. The transmittance value of native rice starch was 35.27%. This is due to native rice starch molecules disintegrate completely during gelatinization process, then starch molecules remain reorganized [39], which corresponding to setback value of the native rice starch as shown in Table 3. However, GCWSS exhibited significantly lower transmittance value than the native rice starch. The decrease in transmittance value observed in GCWSS can likely be attributed to an increase in amylose content and greater mobility during alcoholic-alkaline treatment, as indicated in Table 1. The alcoholic-alkaline treatment is known to depolymerize amylopectin, resulting in the formation of shorter amylose chains [26], [27], which in turn leads to an increase in amylose content. Starch paste turbidity is influenced by the high amylose content and the mobility of these chains, which promote rapid starch retrogradation [37], [38], [40]. In the case of GCWSS + U samples, the transmittance values range from 37.43% to 40.23%. Specifically, the transmittance value of GCWSS + 30 %U significantly increases to 37.43% compared to GCWSS. It has been reported by Chen, Dai [32] that the molecular weight of starch is positively correlated with turbidity. Moreover, ultrasound treatment disrupts and destroys the inter- and intra-starch molecules formed during the ethanol-alkali process, allowing more amylose to leak out of the granules and leading to a decrease in amylose concentration. As a result, the sonication process produces shorter-chain amylose, which contributes to reduced turbidity [10], [28]. This decrease in turbidity is attributed to a reduction in the retrogradation rate caused by a downward trend in amylose content after ultrasound treatment of rice starch, resulting in a decrease in paste turbidity. The loss of birefringence also contributes to increased clarity of the starch paste [10]. Furthermore, the transmittance value greatly increases with higher ultrasound power. This can be attributed to the formation of rough surfaces with fissures on the starch granules (as shown in Fig. 1) and the disruption of the double-helix structure under higher ultrasonic power treatments [40]. Particularly, GCWSS + 100 %U exhibits the highest transmittance value (40.23%). This indicates that treatment with higher ultrasound power (100% ultrasonic power) can reduce the turbidity of starch paste, which is consistent with the observed amylose content and pasting properties (Table 1, Table 3).

**Table 4:** Turbidity of the native, GCWSS and GCWSS treated with ultrasound treatment.

**Sample**	**%Transmittance**
Native rice starch	35.27 ± 0.59^d^
GCWSS	33.53 ± 0.23^e^
GCWSS + 30 %U	37.43 ± 0.31^c^
GCWSS + 70 %U	38.73 ± 0.57^b^
GCWSS + 100 %U	40.23 ± 0.21^a^

Different letters in the same column indicate significant differences from each other *(p* ≤ 0.05).

### 3.7 Freeze-thaw stability

During freezing, water molecules in starch gels form ice crystals, and starch molecules aggregate. When thawed, the starch gels are separated into water and starch-rich phases, this phenomenon is defined the syneresis. Repeating the freeze–thaw cycles enforce the phase separation and ice growth [41]. The extent of phase separation increased with additional freeze–thaw cycles due to an increase in amylopectin retrogradation in the starch-rich phase [42]. Table 5 showed the syneresis of the native rice starch and GCWS starch gels during five freeze–thaw cycles. GCWSS sample presented a higher syneresis with increasing number of freeze-thawing cycles than the native rice starch. The syneresis of the native rice starch was initially 2.81% and increased to 5.29% after fifth freeze-thawing cycles. The syneresis of GCWSS increased 7.90% to 13.77% from the first to the fifth freeze-thawing cycles. A greater syneresis of GCWSS than the native might be related to its high amylose content, the freedom of amylose chains involves the realignment into a more crystalline form during freezing of starch gel. Additionally, GCWSS + U treatment was observed. The higher level of ultrasound power decreased the syneresis of all GCWSS + U samples as compared to GCWSS sample. The maximum syneresis value were presented in 10.62% for GCWSS + 70 %U and 10.55% for GCWSS + 100 %U. This result demonstrated that GCWSS + U was more resistant to syneresis than GCWSS under repeated freeze–thaw cycles which presented in lower percent syneresis between the freeze–thaw cycles. This result might be due to ultrasound treatment breakage of starch chains in amorphous region contributed to reordering of starch chains, therefore a greater number of hydrophilic bonds were exposed which could hold more water during thawing resulting in syneresis decreased [43]. In addition, GCWSS + U exhibited a softer gel texture (as presented in Table 5), which might have less tolerance to the deformation caused during centrifugation resulting in decreased syneresis values.

**Table 5:** Freeze-thaw stability of the native, GCWSS and GCWSS treated with ultrasound treatment.

**Samples**	**Syneresis (%)**				
	**Cycle 1**	**Cycle 2**	**Cycle 3**	**Cycle 4**	**Cycle 5**
Native rice starch	2.81 ± 1.88^a^	2.81 ± 1.87^a^	4.01 ± 0.43^b^	5.09 ± 2.22^a^	5.29 ± 1.99^b^
GCWSS	7.90 ± 1.27^b^	11.47 ± 0.67^a^	11.36 ± 0.76^a^	12.43 ± 1.74^b^	13.77 ± 3.17^b^
GCWSS + 30 %U	7.40 ± 0.80^b^	9.80 ± 2.05^b^	11.94 ± 0.90^b^	11.06 ± 2.34^a^	11.19 ± 2.11^a^
GCWSS + 70 %U	3.34 ± 1.36^a^	4.06 ± 2.10^a^	7.71 ± 1.74^a^	7.86 ± 0.18^a^	7.62 ± 1.47^a^
GCWSS + 100 %U	3.02 ± 3.38^a^	3.32 ± 2.16^a^	6.60 ± 2.30^a^	7.36 ± 1.71^a^	7.53 ± 0.74^a^

Different letters in the same column indicate significant difference from each other *(p* ≤ 0.05).

### 3.8 Gel texture

**Fig. 3:**
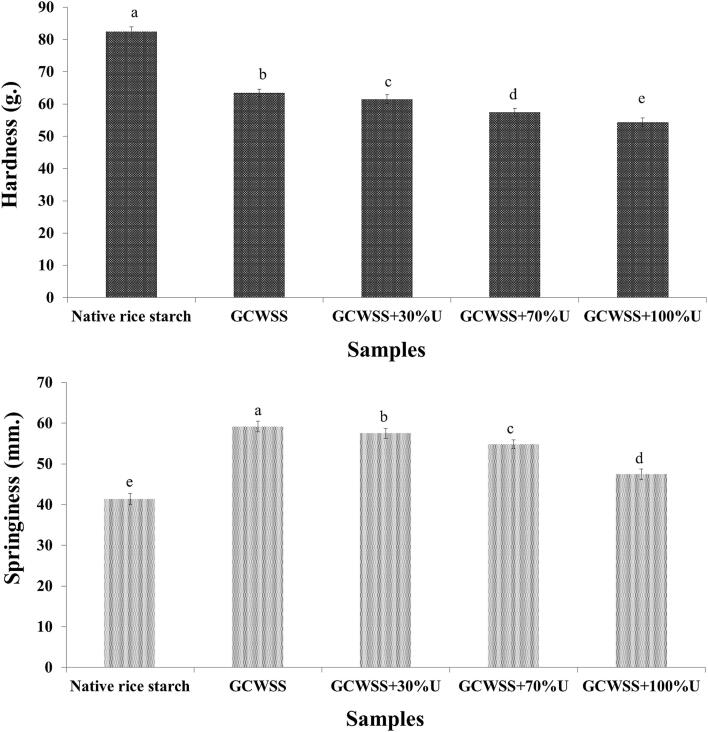
Texture parameters of the native, GCWSS and GCWSS + U samples.

Fig. 3 shows texture parameters of the native rice starch, GCWSS and GCWSS treated with ultrasound treatment. Native rice starch displayed the highest hardness, while the lowest springiness as compared to GCWSS with and without ultrasound treatment. This is because the gel hardness, and springiness were mainly caused by amylose content and retrogradation of starch gel [44], [45], [46]. After alcoholic-alkaline treatment, GCWSS showed significantly lower hardness, while higher springiness than native rice starch. This might be due to alcoholic-alkaline treatment of starch can induce the strongly swollen granules become softer and disintegrate compared to native rice starch (as shown in Fig. 1 and Table 1), consequently decreasing starch hardness, but increasing springiness [44]. Moreover, the hardness of GCWSS coupled with ultrasound treatment was continuously decreased when increasing the ultrasonic power, especially the lowest value was found in the case of treated by 100% ultrasonic power. Additionally, the same trend was found in springiness values of GCWSS + U, however, the springiness of GCWSS + 100 %U was still higher than native starch. This might be due to the reduction of amylose content caused by disruption and destruction of inter- and intra-starch molecule after ultrasound treatment [10], [28], might leads to slower starch retrogradation and softer gel, resulting in decreased hardness as well as changed springiness of the gel structure of GCWSS with ultrasound treatment.

## 4 Conclusion

The solubility and swelling of rice starch is improved when treated with alcohol-alkali treatment and ultrasonic-assisted alcohol-alkali treatment, especially ultrasonic-assisted alcohol-alkali treatment for superior ability. Furthermore, during GWSS treatment brought about changes in the pasting properties, turbidity, cold-water swelling, cold-water solubility, gel texture properties and syneresis value in rice starch. GCWSS + U presented a softer gel and produced clearer gel than GCWSS rice starch. Increasing ultrasound power level enhanced a reduction of syneresis of GCWSS + U. GCWSS + 70 %U and GCWSS + 100 %U showed an increase in the syneresis values occurred at the first freeze–thaw cycle and remained constant after second freeze–thaw cycle. In summary, ultrasound assisted alcohol-alkaline treatment could be used for preparation to produce granular-cold water swelling starch and could be use in frozen products as it had less syneresis and retrogradation in further.

## CRediT authorship contribution statement

**Kannika Kunyanee:** Writing – original draft, Conceptualization, Visualization, Formal analysis, Data curation, Supervision. **Kanyarak Phadtaisong:** . **Jutarat Na Chiangmai:** Formal analysis. **Natch Parittapongsachai:** . **Tai Van Ngo:** Writing – review & editing. **Naphatrapi Luangsakul:** Writing – review & editing. **Sirada Sungsinchai:** Writing – review & editing, Supervision.

## Declaration of Competing Interest

The authors declare that they have no known competing financial interests or personal relationships that could have appeared to influence the work reported in this paper.
